# Asthma control and COPD symptom burden in patients using fixed-dose combination inhalers (SPRINT study)

**DOI:** 10.1038/s41533-019-0159-1

**Published:** 2020-01-07

**Authors:** Nicolas Roche, Vicente Plaza, Vibeke Backer, Job van der Palen, Isa Cerveri, Chelo Gonzalez, Guilherme Safioti, Irma Scheepstra, Oliver Patino, Dave Singh

**Affiliations:** 1 Respiratory Medicine, Cochin Hospital, APHP Centre University of Paris, Institut Cochin (UMR1016), Paris, France; 2 Department of Respiratory Medicine and Allergy, Hospital de la Santa Creu i Sant Pau, Institut d’Investigació Biomédica Sant Pau (IIB Sant Pau), Universitat Autònoma de Barcelona, Department of Medicine, Barcelona, Spain; 3 Department of Respiratory Medicine, Copenhagen University, Copenhagen, Denmark; 4 Department of Research Methodology, Measurement and Data Analysis, University of Twente, Enschede, The Netherlands; 5 Medical School Twente, Medisch Spectrum Twente, Enschede, The Netherlands; 6 Unit of Respiratory Diseases, IRCCS Policlinico San Matteo, University of Pavia, Pavia, Italy; 7 Unit of Statistics and Data Management, Experior SL, 46139 La Pobla de Farnals, Valencia, Spain; 8 Teva Pharmaceutical Industries Ltd, Amsterdam, The Netherlands; 9 Centre for Respiratory Medicine and Allergy, University of Manchester, and the Medicines Evaluation Unit, University Hospital of South Manchester NHS Foundation Trust, Manchester, UK

**Keywords:** Therapeutics, Health care

## Abstract

Previous studies have found suboptimal control of symptom burden to be widespread among patients with asthma and chronic obstructive pulmonary disease (COPD). The Phase IV SPRINT study was conducted in 10 countries in Europe to assess asthma disease control and COPD symptom burden in patients treated with a fixed-dose combination (FDC) of inhaled corticosteroids (ICS) and long-acting beta agonists (LABAs). SPRINT included 1101 patients with asthma and 560 with COPD; all were receiving treatment with an FDC of ICS/LABA, delivered via various inhalers. Data were obtained over a 3-month period, during a single routine physician’s office visit. Asthma control was defined as Asthma Control Test (ACT) score >19. COPD symptom burden was assessed by COPD Assessment Test (CAT), with a CAT score <10 defining low COPD symptom burden. Among patients using any ICS/LABA FDC, 62% of patients with asthma had achieved disease control (ACT score >19) and 16% of patients with COPD had low symptom burden (CAT score <10).

## Introduction

Asthma and chronic obstructive pulmonary disease (COPD) are of significant concern worldwide owing to their high prevalence and substantial clinical and economic burden.^1^ While asthma is the most prevalent respiratory disease in the world (and twice as common as COPD), the mortality rate associated with COPD is eight times higher than for asthma.^1^ The absolute number of patients with these two conditions is increasing as the global population grows.^1^

Inhaled medications are the mainstay of treatment for both COPD and asthma,^2,3^ but many patients with asthma and COPD have poor treatment adherence and/or inhaler technique.^4,5^ Improvements in these patient factors have the potential to substantially increase the effectiveness of therapy and help alleviate disease burden.^6–9^

Combined inhaled corticosteroid (ICS) and long-acting beta agonist (LABA) treatments play a central role in the management of persistent asthma (Step 3 and above)^2^ and have been associated with beneficial effects on lung function, exacerbation rates, and patient-reported outcomes compared with each treatment alone in patients with COPD.^10–12^ Several inhalers containing a fixed-dose combination (FDC) and ICS and LABA are commercially available in Europe for the delivery of maintenance therapies for COPD and/or asthma. The convenience of having both substances in a single inhaler may help improve treatment adherence and is often cost saving, compared with using two separate inhalers.^13^ In April 2014, marketing authorisation was granted for DuoResp^®^ Spiromax^®^, a budesonide/formoterol combination treatment alternative to the existing Symbicort Turbuhaler, which contains the same active substances but uses a different dry powder inhaler (DPI) mechanism with fewer preparation manoeuvres.^14^

The present real-world observational study was conducted to obtain a cross-sectional overview of asthma disease control and COPD symptom burden among patients receiving treatment with an FDC of ICS and LABA administered twice daily using a DPI. Additional aims of the study were to evaluate patient adherence in a real-world clinical practice population and to collect information on the patients’ background and their healthcare utilisation, in order to assess the relationship between these factors and disease burden. Another purpose of the study was to analyse clinical data among patients using the FDC budesonide/formoterol DuoResp Spiromax inhaler. The data on DuoResp Spiromax adherence, satisfaction, and ease of use will be reported separately.

## Results

### Study population

A total of 1138 patients with asthma were enrolled in the study. Enrolment by country is presented in Supplementary Table 1. Among asthma patients receiving treatment with any ICS/LABA, 1101 completed the study. The 4 most frequently used ICS/LABA treatment devices (used by 72.0% of patients) were Symbicort inhalers (298 patients), DuoResp Spiromax inhalers (235 patients), Seretide inhalers (174 patients), and Fostair inhalers (86 patients). Other types of fixed-dose ICS/LABA inhalers were used by 28.0% of patients.

A total of 596 patients with COPD were enrolled in the study. Enrolment by country is presented in Supplementary Table 1. Of these, 560 COPD patients completed the study. The four most frequently used ICS/LABA treatment devices (used by 74.5% of patients) were Seretide inhalers (156 patients), Symbicort inhalers (113 patients), DuoResp Spiromax inhalers (107 patients), and Fostair inhalers (41 patients). Other types of fixed-dose ICS/LABA inhalers were used by 25.5% of patients.

Patient demographics are presented in Table 1a. The mean (standard deviation [SD]) age of the asthma cohort was 53.8 (16.7) years, and asthma was less frequent in men (37.1%) than women (62.9%). The mean (SD) age of the COPD cohort was 69.5 (9.0) years. This group included more men (62.5%) than women (37.5%). Disease characteristics for all patients are presented in Table 1b (additional information regarding previous asthma/COPD treatments is supplied in Supplementary Table 2). The mean (SD) last known forced expiratory volume in 1 s (FEV_1_) % predicted was 81.7% (23.1) in the asthma cohort and 58.3% (23.6) in the COPD group.

**Table 1:** (a) Patient demographic characteristics and (b) clinical characteristics, concomitant conditions, and prior medications.

	Asthma (*N* = 1101)	COPD (*N* = 560)	Total (*N* = 1661)	*p* value
(a)				
Gender, *n* (%)				
Female	693 (62.9)	210 (37.5)	903 (54.4)	<0.001
Male	408 (37.1)	350 (62.5)	758 (45.6)	
Age at study visit, years				
Mean (SD)	53.8 (16.7)	69.5 (9.0)	59.1 (16.3)	<0.001
BMI, kg/m^2^				
Mean (SD)	28.1 (6.1)	27.8 (5.9)	28 (6.0)	0.258
Data unavailable	71	26	97	
Obesity (BMI ≥30), *n* (%)				
Yes	315 (30.6)	161 (30.1)	476 (30.4)	0.906
No	715 (69.4)	373 (69.9)	1088 (69.6)	
Data unavailable	71	26	97	
(b)				
Years since disease diagnosis				
Median (P25, P75)	11 (4, 23)	7 (4, 12)	9 (4, 18)	<0.001
Data unavailable	14	4	18	
FEV_1_, L				
Mean (SD)	2.5 (0.9)	1.5 (0.6)	2.1 (1.0)	<0.001
Data unavailable	186	36	222	
% predicted FEV_1_, %				
Mean (SD)	81.7 (23.1)	58.3 (23.6)	73.1 (25.9)	<0.001
Data unavailable	224	50	274	
Concomitant disease presence, *n* (%)				
Yes	792 (73.1)	459 (83.5)	1251 (76.6)	<0.001
No	292 (26.9)	91 (16.5)	383 (23.4)	
Data unavailable	17	10	27	
Concomitant disease^a^, *n* (%)	(*N* = 1084)	(*N* = 550)	(*N* = 1634)	
Cardiovascular disease	267 (24.6)	269 (48.9)	536 (32.8)	<0.001
Depression or anxiety disorder	126 (11.6)	79 (14.4)	205 (12.5)	0.133
Allergy	304 (28)	26 (4.7)	330 (20.2)	<0.001
Osteoporosis	33 (3.0)	37 (6.7)	70 (4.3)	<0.001
Diabetes	91 (8.4)	84 (15.3)	175 (10.7)	<0.001
Cancer	42 (3.9)	53 (9.6)	95 (5.8)	<0.001
Other	430 (39.7)	276 (50.2)	706 (43.2)	<0.001
Any previous asthma/COPD treatment, *n* (%)				
Yes	575 (56.3)	283 (55.1)	858 (55.9)	0.679
No	446 (43.7)	231 (44.9)	677 (44.1)	
Data unavailable	80	46	126	
Previous asthma/COPD treatment description, *n* (%)	(*n* = 575)	(*n* = 283)	(*n* = 858)	
ICS monotherapy	191 (33.2)	23 (8.1)	214 (24.9)	<0.001
LABA monotherapy	20 (3.5)	21 (7.4)	41 (4.8)	
Fixed-dose combination (different from current)	287 (49.9)	147 (51.9)	434 (50.6)	
Long-acting muscarinic antagonists	13 (2.3)	60 (21.2)	73 (8.5)	
Leukotriene modifier	28 (4.9)	0 (0)	28 (3.3)	
Methylxanthine (theophylline)	1 (0.2)	5 (1.8)	6 (0.7)	
Other	35 (6.1)	27 (9.5)	62 (7.2)	
Data unavailable	0	0	0	
Duration (months) of use of the patient’s current FDC^b^				
Median (P25, P75)	18.7 (8.6, 47.2)	22.4 (10.1, 55.1)	19.7 (9, 50.4)	0.144
Data unavailable	225	130	355	
Disease control (asthma)/Low symptom burden (COPD), *n* (%)				
Yes	684 (62.4)	85 (15.5)	769 (46.7)	<0.001
No	412 (37.6)	464 (84.5)	876 (53.3)	
Data unavailable	5	11	16	

*BMI* body mass index, *COPD* chronic obstructive pulmonary disease, *FDC* fixed-dose combination, *FEV*_*1*_ forced expiratory volume in 1 s, *ICS* inhaled corticosteroid, *LABA* long-acting beta agonist, *SD* standard deviation

^a^Every patient may present several concomitant diseases. Thus percentages have been calculated based on the number of patients

^b^Patients were eligible for the study if they had received a stable dose of ICS/LABA FDC for the 3 months prior to enrolment. One COPD patient who had received treatment for 12 weeks was accepted onto the study by the sponsor

### Primary endpoints

**Fig. 1:**
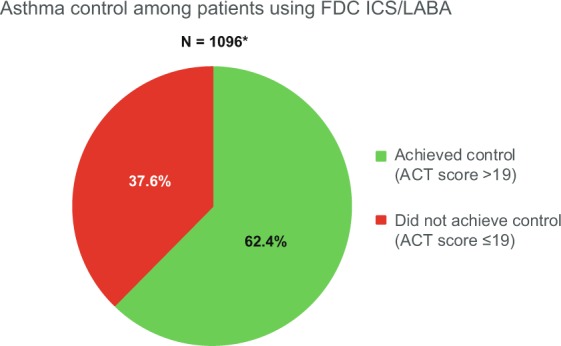
Proportion of asthma patients with disease control, defined as ACT score >19, at the time of assessment after at least 3 months of FDC inhaler use. *Data were unavailable for 5 patients. ACT Asthma Control Test, FDC fixed-dose combination.

**Fig. 2:**
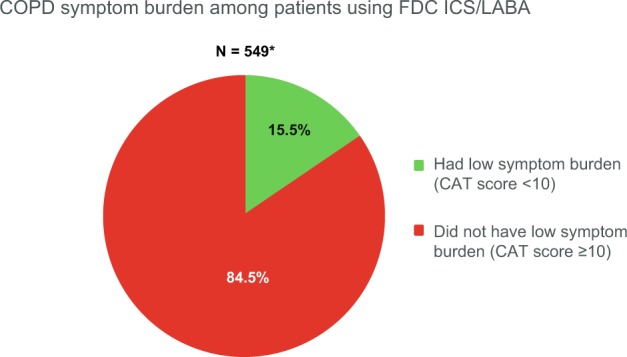
Proportion of COPD patients with low symptom burden, defined as CAT score <10, at the time of assessment, after at least 3 months of FDC inhaler use. *Data were unavailable for 11 patients. CAT COPD Assessment Test, COPD chronic obstructive pulmonary disease, FDC fixed-dose combination.

In the overall asthma population, 684 patients (62.4%) had an Asthma Control Test (ACT) score >19 and were considered to have achieved disease control at the time of the assessment, after ≥3 months of FDC inhaler use (Fig. 1). In the overall COPD population, 85 patients (15.5%) had a COPD Assessment Test (CAT) score <10, defining a low symptom burden at the time of the assessment, after ≥3 months of FDC inhaler use (Fig. 2).

### Secondary endpoints

Table 2 shows an overview of patient adherence to treatment as assessed by Morisky Medication Adherence Scale (MMAS-8^®^) questionnaire. A greater proportion of patients in the COPD group had high adherence compared with patients in the asthma group (51.0% vs 34.4%, respectively). Table 3 provides an overview of health status among SPRINT study participants, based on the EuroQoL 5-dimensional 3-level (EQ-5D-3L) questionnaire findings. Mean (SD) visual analogue scale (VAS) score was greater in patients with asthma than in those with COPD (74.3 [18.3] vs 62.8 [18.5], respectively). The mean (SD) raw EQ-5D-3L index value was also greater in asthma patients than in those with COPD (80.1 [18.5] vs 70.7 [20.5], respectively). Table 4 shows correlations between adherence and health-related quality of life measures. Table 5 shows health-related quality of life measures according to achievement or otherwise of asthma disease control or COPD low symptom burden.

**Table 2:** Adherence to ICS/LABA treatment among SPRINT study participants with asthma and COPD, as assessed by MMAS-8 score and adherence classification.

	Asthma (*N* = 1101)	COPD (*N* = 560)	Total (*N* = 1661)	*p* value
MMAS-8 score				
All ICS/LABA	(*n* = 1101)	(*n* = 560)	(*n* = 1661)	
Median (P25, P75)	7.0 (5.8, 8.0)	8.0 (7.0, 8.0)	7 (5.8, 8)	<0.001
Data unavailable	21	9	30	
MMAS-8 adherence classification, *n* (%)				
All ICS/LABA	(*n* = 1101)	(*n* = 560)	(*n* = 1661)	
High (score = 8)	371 (34.4)	281 (51.0)	652 (40)	<0.001
Medium (6 ≤ score < 8)	382 (35.4)	182 (33.0)	564 (34.6)	
Low (score < 6)	327 (30.3)	88 (16.0)	415 (25.4)	
Data unavailable	21	9	30	

*COPD* chronic obstructive pulmonary disease, *ICS* inhaled corticosteroid, *LABA* long-acting beta agonist, *MMAS-8* 8-item Morisky Medication Adherence Scale

Note: Use of the ©MMAS is protected by US copyright laws. Permission for use is required. A license agreement is available from: Donald E. Morisky, ScD, ScM, MSPH, Professor, Department of Community Health Sciences, UCLA School of Public Health, 650 Charles E. Young Drive South, Los Angeles, CA, 90095-1772

**Table 3:** Health-related quality of life and healthcare utilisation among SPRINT study participants with asthma and COPD.

	Asthma (*N* = 1101)	COPD (*N* = 560)	Total (*N* = 1661)	*p* value
VAS score				
Mean (SD)	74.3 (18.3)	62.8 (18.5)	70.4 (19.1)	<0.001
Data unavailable	26	7	33	
Mean EQ-5D-3L index value				
Mean (SD)	0.80 (0.21)	0.69 (0.23)	0.76 (0.23)	<0.001
Data unavailable	17	4	21	
Number of visits to the doctor/GP due to asthma or COPD				
Mean (SD)	0.4 (0.8)	0.7 (1.2)	0.5 (1)	<0.001
Data unavailable	0	0	0	
Number of emergency department visits due to asthma or COPD				
Mean (SD)	0 (0.2)	0.1 (0.5)	0.1 (0.3)	0.006
Data unavailable	0	0	0	
Number of hospital stays (>1 day) due to asthma or COPD				
Mean (SD)	0 (0.2)	0.1 (0.8)	0.1 (0.5)	<0.001
Data unavailable	0	0	0	

*COPD* chronic obstructive pulmonary disease, *EQ-5D-3L* EuroQoL 5-dimensional 3-level, *SD* standard deviation, *VAS* visual analogue scale

Healthcare resource utilisation in the 3 months preceding the study was determined. In the total cohort, asthma- or COPD-related visits to a physician (median 0, range 0–10) and emergency department (median 0, range 0–6) and hospitalisations lasting >1 day (median 0, range 0–11) were rare events.

## Discussion

The results of this observational study of patients receiving inhaled maintenance treatment with ICS/LABA FDC showed that almost two-thirds of asthma patients met the threshold for asthma control (ACT score >19). The rate of asthma control in the SPRINT study (62.4%) was higher than the pan-European rate reported in the International Cross-Sectional and Longitudinal Assessment on Asthma Control (LIAISON) studies conducted in 2012–2013 (43.5%)^15^ and the 2014 REcognise Asthma and LInk to Symptoms and Experience (REALISE) study conducted across Europe (20%).^16^

Research has shown that asthma patients’ perceptions of disease control are often at odds with more objective measures of disease control. For example, >80% of patients in the REALISE study considered their asthma to be controlled, and more than two-thirds did not consider their condition as serious, whereas in fact only one patient in five met the criteria for controlled asthma.^16^ This underestimation of disease control probably has a significant impact on asthma outcomes. A report by the Royal College of Physicians in the UK estimated that >65% of asthma deaths were associated with potentially preventable factors, including trigger avoidance, medication adherence, and regular medical follow-up.^17^

Comparatively few patients in the SPRINT study (15.5%) met the pre-defined criterion for low COPD symptom burden (CAT score <10). A recently reported multicentre, observational study in Europe classified 21% of COPD patients as ‘controlled’, using the same CAT score criteria.^18^ However, all patients classified had mild-to-moderate COPD. Most patients in the SPRINT cohort were elderly (median age 70 years). As symptoms become more difficult to manage as COPD progresses, the low rate of achievement of the pre-defined CAT score threshold in the SPRINT study COPD cohort is not unexpected. Taken together, these results suggest that a CAT threshold of 10 is probably too low to define low COPD symptom burden.^18,19^ In view of the lack of reversibility that is typical of COPD, the threshold might need to be adapted to the baseline severity of the disease.

Significant negative effects of COPD on health-related quality of life are reflected in the health status scores reported among SPRINT study participants. For example, the mean EQ-5D-3L for a 70-year-old person (mean age of the COPD cohort) living in the European countries that were included in the SPRINT study is between 69.0 and 81.5,^20^ whereas the mean EQ VAS in our COPD cohort was 62.8. Asthma appeared to have less effect on overall health status; the mean EQ VAS in our asthma cohort was 74.3, which is within the average range for a 55-year-old person living in these European countries (72.0–81.7).^20^

The findings of the SPRINT study indicate that suboptimal adherence to inhaled treatment regimens is widespread among patients with asthma and COPD. In particular, only 34.4% of patients with asthma had an MMAS-8 score indicative of high adherence, and >30% had a score indicative of low adherence. Poor adherence leading to poor outcomes in patients with asthma was also seen in the LIAISON study, which used the 4-item Morisky Medication Adherence Scale (MMAS-4) and found that patients with uncontrolled asthma were more likely to have MMAS-4 scores indicative of low adherence than those with controlled asthma.^15^ Consistent with previous reports of relatively inferior adherence among younger vs older patients using inhaled medications,^21^ SPRINT study participants with COPD were more likely than those with asthma to be adherent, with more than half having an MMAS-8 score indicative of high adherence. The rates of adherence and asthma control among participants in the SPRINT study were consistent with other recent reports of real-world studies in both asthma and COPD cohorts.^7,22^ As expected, adherence was markedly lower than is typically seen in clinical trials.

Relationships between disease control and other variables were explored for asthma patients using multiple regression. Although no model could be found that could explain most of the variance in the data, analysis findings suggested that male sex and higher education may be positively associated, and body mass index (BMI) negatively associated, with disease control. These observations are consistent with previous findings.^23,24^

Previous research has linked adherence with clinical symptoms and outcomes.^4,25–27^ However, even in adherent patients, other factors may influence outcomes. For example, inhaler technique is an important determinant of outcome, as it influences the dose of drug delivered to the lungs at each inhalation.^6^ Demographic factors may also influence disease control. For example, the LIAISON study found that females, those aged 40–64 years, and patients who were overweight or obese were less likely to achieve asthma control compared with males, other age groups, and those of normal weight, respectively.^15^ Other studies have confirmed the interaction between obesity and disease control in asthma. Scott et al. showed that, among asthma patients who are adherent to therapy, those who are obese (BMI >30 mg/m^2^) have difficulty achieving asthma control.^28^ Thirty percent of patients in the SPRINT cohort had a BMI >30 kg/m^2^, and the proportion of obese patients was similar in the asthma (30.6%) and COPD (30.1%) cohorts. This is slightly higher than the proportion of obese patients reported in the LIAISON study of European asthma patients (26.1%).^15^

COPD patient demographics showed that participants receiving an ICS/LABA FDC in this study covered the range of COPD subtypes encountered in routine clinical practice (elderly, former or current smokers, high incidence of comorbidity).^29,30^ Owing to the stringent selection criteria that are typically applied, randomised clinical trial cohorts are poorly representative of the broader COPD population, most of whom are treated in primary care.^31,32^ Comorbidities, which may be a reason to exclude patients from randomised clinical trials,^32^ were present in 83.5% of COPD patients in the SPRINT study. This is higher than the prevalence of significant comorbidity reported in a Norwegian study of COPD patients seen in primary care or hospital outpatient clinics (60.7%)^32^ but lower than the prevalence reported in US COPD patients (96.4%), according to the National Health and Nutrition Examination Survey, 1999–2008.^30^ Furthermore, the mean age of COPD patients in the SPRINT study, 69.5 years, was markedly higher than is typical for large randomised COPD studies, for which Kruis and colleagues reported a mean age of 63.7 years.^31^

Strengths of the SPRINT study include the pan-European context, involving 10 countries from across Europe, and the generalisability to the population of patients seen in everyday clinical practice. The study included all patients with asthma or COPD receiving twice-daily ICS/LABA FDC and did not exclude anyone on demographic or clinical grounds. Another strength is the inclusion of patient-reported outcome measures that are widely used and validated in COPD and asthma populations.^33^ However, the SPRINT study also has some limitations. As an observational study, it is subject to confounders and potential bias. For example, assessments by patients were subject to recall bias and reporting bias (e.g. with regard to adherence). Wherever possible, attempts were made to minimise recall bias by using patient records to assess preceding medical visits. In addition, this study was not designed to assess the comparative effectiveness of different ICS/LABA FDC inhalers, and the findings likely describe a class effect rather than the effect of any specific type of inhaler.

The results of this observational clinical study showed that the majority (62%) of asthma patients using ICS/LABA FDC inhalers meet the threshold for asthma control in real-world clinical practice and that the burden of symptoms for COPD in real-world clinical practice is high. Adult patients with asthma and COPD have a high rate of comorbidities, but adherence to ICS/LABA therapy in real-world asthma and COPD patients is low.

In conclusion, this large, observational study in adult patients with asthma and COPD across 10 countries in Europe found that more than half of asthma patients using any ICS/LABA FDC inhaler met the threshold for asthma disease control. The pre-defined threshold for low symptom burden was met by few patients with COPD; however, this is probably to be expected in a chronic disease with an overall more advanced patient population than for asthma. Suboptimal adherence was widespread, particularly among asthma patients. Both patient populations had a high rate of comorbidities, and many of the patients would have been ineligible to participate in recent randomised controlled trials of asthma and COPD medications. The findings of the SPRINT study highlight the need for more attention by European physicians on patient-reported symptoms and adherence in the asthma and COPD patients they see in clinical practice.

## Methods

### Study design and participants

This was a Phase IV, multi-country, observational, prospective study in patients with asthma or COPD who were on a stable dose of ICS/LABA FDC administered by DPI twice daily. The study was performed between May 2015 and April 2017 at 140 centres in Croatia (15 sites), Denmark (7), Ireland (7), Italy (25), The Netherlands (7), Norway (4), Portugal (10), Spain (39), Sweden (2), and the UK (24). Prior to study initiation, Independent Ethics Committee approval was obtained from all relevant national or regional bodies.

Data were collected for the whole population of patients receiving any ICS/LABA treatment in a routine setting (on a single occasion during an otherwise normal visit to a physician’s office or clinic). Participating physicians did not perform any additional medical procedures, other than those that were part of routine practice. All treatment decisions were at the sole discretion of participating physicians.

### Patients

Patients were eligible to participate if they had a diagnosis of persistent asthma or COPD in accordance with the GINA or GOLD reports, respectively, and were receiving treatment with a stable dose of an ICS/LABA FDC, administered twice daily by DPI for 3 months prior to enrolment. Dose stability was defined as no change in dose by >50% in the past 3 months. Patients with asthma were eligible to participate if they were aged ≥18 years, and those with COPD could participate if they were aged ≥40 years and/or a current or ex-smoker with a smoking history of >10 pack-years. In addition, all participants had to agree to participate in the study and to disclose any medical events to the treating physician and to be willing and able to provide written informed consent and complete the questionnaires. The only exclusion criterion was that patients could not currently be, or plan to be, enrolled in an interventional study.

### Assessments

Demographic and clinical characteristics (including medical history, previous and concomitant respiratory medications, number of exacerbations in the previous year, and healthcare utilisation in the past 3 months) were collected from the patient’s medical records by the investigational centres. During the study visit, the patient’s respiratory disease burden was evaluated by a series of paper questionnaires, completed by patients in their local language. Asthma disease control was assessed using the ACT questionnaire.^34^ The burden of COPD symptoms was evaluated using CAT.^35^ Patients rated their own adherence to therapy using the MMAS-8 questionnaire.^36–38^ Patients also rated their general health status using the EQ-5D-3L questionnaire.^39^ The two-part questionnaire is comprised of the EQ-5D descriptive system and the EQ VAS, which measure 5 dimensions (mobility, self-care, usual activities, pain/discomfort, anxiety/depression) on a 3-point scale and self-rated health on a VAS, respectively.

### Endpoints

The primary endpoint was the percentage of patients whose respiratory disease was well controlled, which was defined as having an ACT score >19 in patients with asthma and low symptom burden (defined as CAT score <10) in patients with COPD. Secondary endpoints were self-reported adherence based on the MMAS-8 score, health status defined by the EQ-5D-3L, and recent healthcare resource utilisation in terms of physician visits, emergency room visits, and hospitalisations lasting >1 day for asthma or COPD.

### Statistical analyses

A target sample size of approximately 792 patients with asthma and 648 patients with COPD was planned. Based on the calculated sample size, the half-width of the 95% confidence interval for the proportion of patients with asthma with controlled disease is 0.035 for all patients treated with ICS/LABA. Similarly, based on this sample size, the half-width of the 95% confidence interval for the proportion of patients with COPD with low symptom burden (considered controlled disease) is 0.032 for all patients treated with ICS/LABA. Statistical analysis was based on the Safety Set, which included all patients enrolled in the study in accordance with the inclusion criteria.

All data (including demographic characteristics, medical history, previous and concomitant respiratory medications, primary and secondary outcome variables, number of exacerbations in the previous year, and healthcare utilisation in the past 3 months) were summarised descriptively using number and percentage for categorical variables and mean (SD) or median and 25th and 75th percentiles, as appropriate, for continuous variables.

Test parameters for hypothesis testing are summarised in Supplementary Table 3. Chi-square test (or Fisher’s exact test when applicable) was used for comparisons between categorical variables. Comparison of continuous variables between two groups (e.g. cohorts) was performed by means of *t* test. Hypotheses for *t* test were assessed using Kolmogorov–Smirnov test with Lilliefors correction (normality) and Levene test (homoscedasticity). If homoscedasticity failed, Welch *t* test was considered instead; if normality failed, Wilcoxon test was considered, whether homoscedasticity failed or not. Pearson correlation was considered to assess the relationship between two continuous variables. If normality failed, Spearman and Kendall correlation were obtained instead. Unless otherwise stated, *p* values were two sided. For both the chi-square test and Fisher’s exact test, Phi was obtained as a measure of the effect size for 2 × 2 tables and Cramer’s *V* for tables with more than 2 rows. For the *t* test and Wilcoxon test, Cohen’s *d* and Cliff’s delta were provided, respectively, as a measure of the effect size.

**Table 4:** Correlation between adherence and health-related quality-of-life measures.

Correlation analyses	Correlation estimate	*p* value^a^
Adherence (MMAS-8 score) and VAS score	(*N* = 1603)	
Spearman correlation	−0.081	0.001^b^
Kendall correlation	−0.061	0.001
Adherence (MMAS-8 score) and EQ-5D-3L index value	(*N* = 1616)	
Spearman correlation	−0.085	0.001
Kendall correlation	−0.065	0.001

Note: Use of the ©MMAS is protected by US copyright laws. Permission for use is required. A license agreement is available from: Donald E. Morisky, ScD, ScM, MSPH, Professor, Department of Community Health Sciences, UCLA School of Public Health, 650 Charles E. Young Drive South, Los Angeles, CA, 90095-1772

*MMAS*-8 8-item Morisky Medication Adherence Scale

^a^For hypothesis contrast, null hypothesis was correlation = 0 and alternative hypothesis was correlation different from 0

^b^*p* value for Spearman correlation could not be computed exact due to ties

**Table 5:** Health-related quality of life and disease control.

Disease control	Yes (*N* = 769)	No (*N* = 876)	Total (*N* = 1645)	*p* value
VAS score				
Mean (SD)	80.1 (15)	62.1 (18.4)	70.5 (19.1)	<0.001
Data unavailable	18	15	33	
Mean EQ-5D-3L index value				
Mean (SD)	0.9 (0.2)	0.7 (0.2)	0.8 (0.2)	<0.001
Data unavailable	15	6	21	

In an exploratory analysis, multiple regression models were constructed to analyse the relationship between the dependent variable “disease control” variable (based on ACT or CAT scores) and several independent variables (age, gender, BMI, smoking, education, adherence, and comorbidity).

At the end of the study, all data were pooled, and global analysis performed (additional analyses for each country, and intercountry differences were also performed in a post hoc exploratory analysis). No imputation was made for missing data.

### Ethics approval

The study protocol was approved by the Hospital Universitario de Canarias, Spain; Lakemedelsverket and the National Ethics Committee, Sweden; 25 local Ethics Committees in Italy; The Foundation for the Code for Pharmaceutical Advertising, Netherlands; 10 local Ethics Committees in Portugal; one National Ethics Committee in the UK; one National Ethics Committee in Ireland; and one National Ethics Committee in Croatia.

### Reporting summary

Further information on research design is available in the Nature Research Reporting Summary linked to this article.

## Supplementary information

Supplementary Material

Reporting Summary

## Data Availability

Data are available upon reasonable request; please contact oliver.patino@tevaeu.com with any enquiries regarding data access.
