# Supplementary material for: Asthma control and COPD symptom burden in patients using fixed-dose combination inhalers (SPRINT study)
Source: NPJ Prim Care Respir Med. 2020 Jan 7;30:1. doi: 10.1038/s41533-019-0159-1 (PMC6946676; doi:10.1038/s41533-019-0159-1)
Supplement: Supplementary file 1 — Supplementary Material [file 41533_2019_159_MOESM1_ESM.pdf]

Supplementary Table 1. Patients enrolled in the study by country

Country	Asthma (N = 1138)	COPD (N = 596)
Spain	211	64
Denmark	110	37
Norway	25	8
Sweden	21	23
Italy	211	111
The Netherlands	102	83
Portugal	85	34
UK	208	140
Ireland	20	23
Croatia	145	73

COPD chronic obstructive pulmonary disease

Supplementary Table 2. Previous asthma/COPD treatment according to ATC code[1], n (%)

ATC code	Asthma (N = 575)	COPD (N = 283)	Total (N = 858)
R01AD	1 (0.2)	0 (0)	1 (0.1)
R03AC	41 (7.1)	26 (9.2)	67 (7.8)
R03AK	285 (49.6)	144 (50.9)	429 (50)
R03AL	1 (0.2)	6 (2.1)	7 (0.8)
R03BA	208 (36.2)	27 (9.5)	235 (27.4)
R03BB	15 (2.6)	80 (28.3)	95 (11.1)
R03DA	1 (0.2)	5 (1.8)	6 (0.7)
R03DC	28 (4.9)	1 (0.4)	29 (3.4)
R03DX	2 (0.3)	0 (0)	2 (0.2)

ATC Anatomical Therapeutic Chemical Classification System, *COPD* chronic obstructive pulmonary disease

[1]Patients may have received treatments coded with more than one ATC code. Percentages have been calculated based on the number of patients receiving any previous treatment.

Supplementary Table 3. Test parameters for hypothesis testing of variables among SPRINT study participants with asthma and COPD

	Test parameters					
	X^2	W	t	df	95% CI	Effect size
Patient demographic and clinical characteristics						
Gender, n (%)						
Female<br>Male	95.831	-	-	1	18.5, 27.5	0.24
Age at study visit, years						
Mean (SD)	-	135808	-	-	-16.0, -13.0	-0.56
BMI, kg/m^2						
Mean (SD)<br>Data unavailable	-	-	1.131	1562	0.99, 1.00	0.06
Obesity (BMI ≥30), n (%)						
Yes<br>No	0.014	-	-	1	-4.8, 5.7	0
Years since disease diagnosis						
Median (P25, P75)<br>Data unavailable	-	370598	-	-	2.0, 4.0	0.23
FEV_1, L						
Mean (SD)<br>Data unavailable	-	-	25.298	1437	1.79, 1.95	1.39
% predicted FEV_1, %						
Mean (SD)<br>Data unavailable	-	-	18.124	945.92	8.6, 11.2	1.01
Concomitant disease presence, n (%)						
Yes<br>No<br>Data unavailable	21.382	-	-	1	-18.1, -7.7	0.11
Concomitant disease, n (%)						
Cardiovascular disease	96.469	-	-	1	-29.7, -19.5	0.24
Depression or anxiety disorder	2.253	-	-	1	-12.9, 1.8	0.04
Allergy	121.641	-	-	1	28.2, 36.4	0.27
Osteoporosis	11.189	-	-	1	-32.7, -7.4	0.08
Diabetes	17.337	-	-	1	-24.2, -8.0	0.1
Cancer	21.081	-	-	1	-34.3, -12.7	0.11
Other	16.011	-	-	1	-14.3, -4.8	0.1
Any previous asthma / COPD treatment, n (%)						

Yes	0.172	-	-	1	-3.8, 6.0	0.01
No						
Data unavailable						
Previous asthma/COPD treatment description, n (%)						
ICS monotherapy	-	-	-	-	-	0.43
LABA monotherapy						
Fixed dose combination (different from current)						
Long-acting muscarinic antagonists						
Leukotriene modifier						
Methylxanthine (theophylline)						
Other						
Data unavailable						
Duration (months) of use of the patient's current FDC						
Median (P25, P75)	-	178974	-	-	-3.5, 0.4	-0.05
Data unavailable						
Disease control (asthma) / Low symptom burden (COPD), n (%)						
Yes	321.672	-	-	1	37.8, 46.0	0.44
No						
Data unavailable						
Adherence to ICS/LABA treatment						
MMAS-8 score						
All ICS/LABA						
Median (P25, P75)	-	229104	-	-	-0.5, 0	-0.23
Data unavailable						
MMAS-8 adherence classification, n (%)						
All ICS/LABA						
High (score = 8)	55.219	-	-	2	NA	0.18
Medium (6≤ score <8)						
Low (score <6)						
Data unavailable						
Health-related quality of life and healthcare utilisation						
VAS score						
Mean (SD)	-	406120	-	-	10, 15	0.37
Data unavailable						
Mean EQ-5D-3L index value						
Mean (SD)	-	384424	-	-	0.1, 0.1	0.28
Data unavailable						
Number of visits to the doctor / GP due to asthma or COPD						
Mean (SD)	-	268081	-	-	0, 0	-0.13

Data unavailable						
Number of emergency department visits due to asthma or COPD						
Mean (SD)<br>Data unavailable	-	299263	-	-	0, 0	-0.03
Number of hospital stays (> 1 day) due to asthma or COPD						
Mean (SD)<br>Data unavailable	-	295381	-	-	0, 0	-0.04
Health-related quality of life and disease control						
VAS score						
Mean (SD)<br>Data unavailable	-	506801	-	-	16, 20	0.57
Mean EQ-5D-3L index value						
Mean (SD)<br>Data unavailable	-	492052	-	-	0.2, 0.2	0.5

BMI body mass index, *CI* confidence interval, *COPD* chronic obstructive pulmonary disease, *df* degrees of freedom, *EQ-5D-3L* EuroQoL 5-dimensional 3-level, *FDC* fixed-dose combination, FEV_1 forced expiratory volume in 1 second, *GP* general practitioner, *ICS* inhaled corticosteroids, *LABA* long-acting beta-agonist, *MMAS-8* Morisky Medication Adherence Scale, *NA* not applicable, *P25* 25th percentile, *P75* 75th percentile, *SD* standard deviation, *t* t-test, *VAS* visual analogue scale, *W* Wilcoxon test, X^2 chi square

Note: Use of the ©MMAS is protected by US copyright laws. Permission for use is required. A license agreement is available from: Donald E. Morisky, ScD, ScM, MSPH, Professor, Department of Community Health Sciences, UCLA School of Public Health, 650 Charles E. Young Drive South, Los Angeles, CA, 90095-1772.
